# Neutrophil Extracellular Traps and Interleukin 17 in Ankylosing Spondylitis

**DOI:** 10.31138/mjr.32.2.182

**Published:** 2021-06-30

**Authors:** Charalampos Papagoras, Akrivi Chrysanthopoulou, Alexandros Mitsios, Victoria Tsironidou, Konstantinos Ritis

**Affiliations:** 1 First Department of Internal Medicine, University Hospital of Alexandroupolis, Democritus University of Thrace, Alexandroupolis, Greece; 2 Laboratory of Molecular Hematology, Democritus University of Thrace, Alexandroupolis, Greece

**Keywords:** Ankylosing spondylitis, neutrophil extracellular traps, interleukin 17

## Abstract

Ankylosing spondylitis (AS) is a chronic inflammatory disease traditionally regarded as mediated by T lymphocytes. Recent progress has identified that cells of innate immunity are also important for the processes of inflammation and new bone formation, a hallmark of AS. Moreover, interleukin-17 (IL-17) is a cytokine implicated in both processes. Neutrophils are increasingly recognized as mediators of autoinflammatory and autoimmune diseases through several mechanisms, one being the release of neutrophil extracellular traps (NETs). NETs are equipped with an array of bioactive molecules, such as IL-1β or IL-17. It appears that the molecules expressed over NETs vary across different disorders, reflecting diverse pathophysiologic mechanisms. As few studies have investigated the role of neutrophils in AS, the purpose of this research protocol is to study whether neutrophils from AS patients are more likely to form NETs, whether IL-17 and IL-1β are expressed over those NETs and if NETs affect new bone formation.

## INTRODUCTION

Ankylosing spondylitis (AS) is a chronic systemic inflammatory disease affecting both musculoskeletal and extraskeletal structures, such as the intestine, the skin, and the uvea.^1^ The musculoskeletal manifestations characteristically involve the spine, which is inflamed typically at the vertebral corners, as well as the apophyseal joints. The inflammation results in bone erosion ultimately followed by an aberrant reparative process, whereby neoosteogenesis, syndesmophyte formation, and bony ankylosis take place.^2^ The fundamental pathogenetic process is considered to be enthesitis, ie, the inflammation of the attachment site to the bone of force-conveying fibrous structures, such as tendons and ligaments, the most typical being the enthesis of Achilles tendon.^1^

Due to the strong association between AS and the HLA B27 molecule, which, by operating as a major histocompatibility class II molecule, participates in the cognate activation of CD8+ positive T cells, AS was initially considered as an autoimmune disease driven by the inadvertent recognition of auto- or allo-antigens. However, evidence for the existence of such (auto)antigens is still poor. In contrast, alternative theories have suggested that HLA B27 may induce the activation of the immune system through non-cognate mechanisms, such as by forming aberrant structures, eg, misfolded or homodimeric complexes.^3–5^ Indeed, innate cells, such as innate lymphoid T cells (ILC), natural killer (NK) cells, CD14+ myeloid cells, and monocytes seem to play an important role in AS pathogenesis. Those cells mediate their effects through the secretion of cytokines, such as IL-23, IL-17, IL-1β, IL-22 and tumour necrosis factor-α (TNFα). ^6–10^

Neutrophil is a cell type of key importance for the innate immunity: it is capable of sensing danger signals, reacting swiftly against pathogens and delivering signals to other cell types during the inflammatory process. Those functions are achieved through receptors, such as toll-like receptors (TLR), phagocytosis, degranulation and release of bioactive substances, such as enzymes, and, finally through a particular type of cell death, whereby the cell releases its chromatin in the form of a mesh, called neutrophil extracellular traps (NETs).^11^ Over NETs molecules like IL-1β, IL-17, tissue factor or the antimicrobial protein LL-37 are often detected, which remain biologically active even following neutrophil death.^12–19^

NETs are involved in the pathogenesis of autoinflammatory diseases, like familiar Mediterranean fever (FMF) or adult-onset Still’s disease (AOSD). During an FMF attack, neutrophils release NETs carrying IL-1β, which further sustains inflammation.^12–13^ Besides, IL-1β blockade with anakinra or canakinumab are to date the most effective treatment of FMF, while similar observations have been published for AOSD as well.^14–15^ Even in classic autoimmune disorders, like systemic lupus erythematosus and ANCA-associated vasculitides, NETs appear to boost the autoimmune process by providing autoantigens and acting as a prothrombotic and proinflammatory machinery, exposing tissue factor, pro-inflammatory cytokines or modifying the function of other immune cells, such as dendritic cells, or non-immune cells, such as fibroblasts.^18–22^ In conclusion, NETosis possibly represents a generic mechanism of final neutrophilic reaction, which may vary in its details depending on the particular disorder. That is, a different set of molecules decorate NETs in each condition reflecting the neutrophil transcriptional process as it had been configured by the cell’s microenvironment before NETosis occurred.^23^

Much less is known about neutrophils and NETs in the Spondyloarthritides (SpA). In the inflammatory bowel disease (IBD), a condition in which axial SpA is clinically manifest in almost 10% of patients, neutrophils show diverse responses as far as NET formation is concerned. In the case of ulcerative colitis IL-1β bearing NETs are formed, while in Crohn’s disease no NETosis is observed.^24^

The peripheral arthritis of SpA is characterized by the presence of IL-17 in the synovial fluid and the synovium itself, in which it is mainly expressed within mast cells and neutrophils, but not T cells.^25^ Notably, IL-17-containing neutrophils have been identified in vertebral biopsies of AS patients, representing the major IL-17-expressing cell type.^26^ On the other hand, the role of IL-17 in AS has gained much interest in the recent years. While it was initially thought to be produced by a particular type of T helper cells (TH17), nowadays the role of IL-17-expressing cell types of innate immunity is gradually uncovered and includes type 3 innate lymphoid cells (ILC3), γ/δ Τ cells and ΝΚ cells.^27^

Moreover, IL-17 appears to be a cytokine involved not only in inflammation and bone erosion through RANKL upregulation, but also in new bone formation.^27^ However, the signals eliciting IL-17 release in the axial skeleton from the various cell types, as well as the sequence of events leading from IL-17 expression up to new bone formation, still remain unclear. The aim of the current protocol is to investigate the role of neutrophils in AS, particularly whether they form NETs, whether those NETs carry IL-17 or other bioactive molecules, as well as their biological effects on both aspects of AS, inflammation and bone metabolism.

## MATERIALS AND METHODS

This is a prospective study that will be performed at the University Hospital of Alexandroupolis. Patients with AS, as well as non-AS control volunteers will be asked to participate in the study after giving their written informed consent. The study protocol has been approved by the Ethics Committee of the University Hospital of Alexandroupolis and the study will conform with the tenets of the Declaration of Helsinki.

Peripheral blood (~20ml) will be collected from patients with active AS, patients with AS in remission/low disease activity and healthy donors. The estimated number of participants is 10 for either patient group and 20 for controls. Peripheral neutrophils will be isolated for immediate functional assays or stored for subsequent experiments. Serum will also be collected. For the bone formation experiments, part of bone marrow aspirates drawn for diagnostic purposes from patients being evaluated for anaemia (in the absence of neoplasia or systemic inflammation) will also be collected in order to obtain mesenchymal stem cells (MSCs)

The following clinical variables of AS patients will be recorded: year of birth, sex, height, weight, year of AS symptom onset, year of AS diagnosis, presence of peripheral arthritis, enthesitis, dactylitis, psoriasis, IBD, uveitis, carriage of HLA B27, presence of spinal syndesmophytes, treatment (non-steroidal anti-inflammatory drugs, synthetic disease-modifying anti-rheumatic drugs, glucocorticoids, biologic agents), measures of disease activity (erythrocyte sedimentation rate, C-reactive protein, BASDAI, ASDAS, patient’s global evaluation of disease activity on a visual analogue scale). Levels of disease activity will be characterised according to currently accepted ASDAS cut-offs.

### Presence of NETs in AS

As an initial step to show whether NETs are involved in AS pathogenesis, neutrophils from patients with active AS will be cultured for 3 hours and subsequently stained with markers of neutrophils (neutrophil elastase, NE) and NET formation (citrullinated histone-3, citH3). DNA will be stained with DAPI. Observation will be performed with immunofluorescence microscopy.

For quantification of NET release, AS patient neutrophils will be cultured in appropriate medium for 4 hours and then NET structures will be isolated with intense shaking. MPO/DNA complex ELISA will subsequently be performed, which expresses NET release in a semi-quantitative manner. MPO/DNA complex ELISA will also be performed directly on the serum of AS patients. Healthy donor neutrophils and serum will be used as control in all the above experiments.

### Protein content of AS NETs

The presence of IL-17A and IL-1β (a key cytokine in most autoinflammatory disorders) over AS-derived NETs will be examined with immunofluorescence using appropriate markers (staining for IL-17A/NE/DAPI or IL-1β/NE/DAPI) and isotype controls. To verify the presence of IL-17 or IL-1β, AS neutrophils will be allowed to form NETs in culture and then NET proteins will be collected following NET digestion with a DNase-I. Interleukin-17 and IL-1β protein will be expressed semiquantitavely with immuno-blotting, while ELISA will be performed to quantify IL-17 and IL-1β over NETs. The expression of the *IL-17A* gene in AS neutrophils will be investigated by quantifying IL-17A mRNA using qPCR. The above experiments will be controlled using healthy donor neutrophils.

### Ability of AS inflammatory microenvironment to induce NETs

Healthy donor neutrophils will be incubated in the presence of serum from AS patients or healthy donors and their ability to form NETs will be examined directly with immunofluorescence, as well as with MPO/DNA complex ELISA applied on NET structures. Further, the presence of IL-17A and IL-1β over those NETs will also be examined directly with immunofluorescence and with ELISA applied on NET structures. In order to examine the importance of AS inflammatory milieu, the above experiments will be repeated with the additional blockade of the effects of cytokines, particularly IL-1, IL-23 and TNFα.

### Effect of the AS inflammatory microenvironment or NETs on the differentiation of bone marrow mesenchymal stem cells

The bone marrow mesenchymal stem cells (MSCs) are multipotent cells, which, depending on their micro-environment, may differentiate towards osteoblasts, chondrocytes or adipocytes. We will examine whether AS microenvironment or neutrophils have any effect on the proliferation and differentiation of MSCs. For this purpose, MSCs isolated from bone marrow aspirates from non-AS patients will be cultured in the presence of AS-derived serum or AS-derived NETs. Healthy serum and NETs formed by healthy donor neutrophils treated with ionomycin (a generic NET inducer) will serve as controls. The differentiation of MSCs towards bone forming cells will be assessed with special stains (Alizarin Red, von Kossa) in inverted microscope, as well as quantifying mRNA expression of genetic markers with qPCR, such as alkaline phosphate, osteocalcin, distal-less homeobox protein 5 and runt-related transcription factor 2.

## IMPORTANCE OF THE STUDY

Ankylosing spondylitis is a chronic inflammatory disease, causing pain, limiting patient function due to spinal inflammation, and producing chronic disability due to bony ankylosis. Despite recent progress, the core question on the sequence of events leading from inflammation to new bone formation remains unanswered. Although modern biological therapies are effective in suppressing inflammation, high response levels are achieved by less than a half of patients.^28^ Moreover, no treatment has been convincingly proved to halt structural damage so far.^29^ Given that the disease affects people during their most productive age, there is a great need to clarify the mechanisms of spinal inflammation and ankylosis, in order to optimize treatments and identify new therapeutic targets. There is plenty of evidence that in the pathophysiology of AS-related inflammation innate immunity mechanisms are at least as important as acquired immunity. Studies in other diseases have shown that neutrophils may express IL-17 and that, through NET formation, it may play a role in the initiation and prolongation of inflammation, but also in the modification of the function of non-immune cells in its vicinity. This study will be the first systemic investigation of neutrophils in AS aiming at answering several research questions:

- Whether neutrophils in AS show increased NET formation
- Whether NET formation parallels disease activity
- Whether AS-derived NETs carry IL-17 and/or IL-1β
- The factors affecting NET formation in AS
- Whether NETs affect the phenotype of MSCs and, particularly, if they promote differentiation toward bone forming cells
